# Epidemic and Evolutionary Characteristics of Swine Enteric Viruses in South-Central China from 2018 to 2021

**DOI:** 10.3390/v14071420

**Published:** 2022-06-28

**Authors:** Chang Li, Hongyu Lu, Chao Geng, Keli Yang, Wei Liu, Zewen Liu, Fangyan Yuan, Ting Gao, Shuangshuang Wang, Ping Wen, Haofei Song, Yongxiang Tian, Danna Zhou

**Affiliations:** 1 Key Laboratory of Prevention and Control Agents for Animal Bacteriosis (Ministry of Agriculture and Rural Affairs), Institute of Animal Husbandry and Veterinary, Hubei Academy of Agricultural Sciences, Wuhan 430064, China; lichang1113@hbaas.com (C.L.); luhongyu053901@163.com (H.L.); a18163131821@163.com (C.G.); keliy6@hbaas.com (K.Y.); liuwei@hbaas.com (W.L.); liuzwen2004@hbaas.com (Z.L.); fyyuan@hbaas.com (F.Y.); gaoting2017@hbaas.com (T.G.); wss20220422@163.com (S.W.); 18229001077@163.com (P.W.); 17861509838@163.com (H.S.); 2 Hubei Provincial Key Laboratory of Animal Pathogenic Microbiology, Wuhan 430064, China

**Keywords:** swine enteric viruses, porcine epidemic diarrhea virus, epidemic and evolutionary characteristics, south-central China

## Abstract

Swine enteric viruses are a major cause of piglet diarrhea, causing a devastating impact on the pork industry. To further understand the molecular epidemiology and evolutionary diversity of swine enteric viruses, we carried out a molecular epidemiological investigation of swine enteric viruses (PEDV, PDCoV, PoRVA, and TGEV) on 7107 samples collected from pig farms in south-central China. The results demonstrated that PEDV is the predominant pathogen causing piglet diarrhea, and its infection occurs mainly in relatively cold winter and spring in Hunan and Hubei provinces. The positive rate of PEDV showed an abnormal increase from 2020 to 2021, and that of PoRVA and PDCoV exhibited gradual increases from 2018 to 2021. PEDV-PoRVA and PEDV-PDCoV were the dominant co-infection modes. A genetic evolution analysis based on the PEDV S1 gene and ORF3 gene revealed that the PEDV GII-a is currently epidemic genotype, and the ORF3 gene of DY2020 belongs to a different clade relative to other GII-a strains isolated in this study. Overall, our results indicated that the variant PEDV GII-a is the main pathogen of piglet diarrhea with a trend of outbreak. G9 is the dominant PoRVA genotype and has the possibility of outbreak as well. It is therefore critical to strengthen the surveillance of PEDV and PoRVA, and to provide technical reserves for the prevention and control of piglet diarrhea.

## 1. Introduction

Swine diarrhea is a pathological symptom caused by multi-factor disorders, including pathogenic microorganisms, feeding environment, and the immune level. Viral diarrhea is a prevalent form of piglet diarrhea in farms, which is mainly manifested as anorexia, vomiting, watery diarrhea, and then body failure and even death [1]. At present, the most common viral pathogens causing piglet diarrhea include porcine epidemic diarrhea virus (PEDV), porcine Group A rotarvirus (PoRVA), porcine deltacoronavirus (PDCoV), transmissible gastroenteritis virus (TGEV), etc. [2]. Both PEDV and TGEV have caused the pandemic of swine diarrhea [3,4].

PEDV can cause acute contact infectious diseases characterized by watery diarrhea and dehydration. Pigs of all ages are susceptible to PEDV, particularly suckling piglets within 7 days of age, which tend to have the most severe symptom with an infection fatality rate up to 90–100% [5]. The clinical symptoms caused by TGEV are similar to those caused by PEDV. TGEV mainly infects piglets within 10 days of age, and the infection rate has been maintained at a relatively low level in recent years [6,7]. PoRVA causes diarrhea, vomiting, and other symptoms, and mainly infects piglets within 60 days of age [8]. PoRVA is distributed worldwide, and its prevalence has shown a steady upward trend in these years, which has aroused the attention of many countries. Moreover, the PoRVA is also a main cause of severe diarrhea in human infants and other young animals through zoonotic transmission [9,10,11]. It has been reported that rotavirus infection kills between 454,000 and 705,000 children under the age of 5 each year in developing countries [12].

After 2010, large-scale outbreaks of porcine epidemic diarrhea (PED) have frequently occurred worldwide. Etiological studies have demonstrated that the PEDV epidemic strains that cause this epidemic have greater genetic variations than the PEDV vaccine strain CV777. The homology of the S gene between the PEDV epidemic strains and CV777 is only 91% to 94% [5,13]. With the commercialization of the PEDV-TGEV inactivated vaccine, PEDV-TGEV-PoRVA (G5 type) live vaccine, and some other antiviral products [14,15,16,17,18], the PEDV epidemic has been better controlled. However, mutation still frequently occurs in the PEDV gene. Therefore, it is highly necessary to monitor the PED epidemic trend and PEDV gene mutation for the timely prevention and control of the epidemic [14,18]. In this study, we conducted an epidemiological investigation and genetic evolution analysis of swine enteric viruses in major pig-producing provinces in China, aiming to clarify the genetic variation trend of PEDV and PoRVA epidemic strains in recent years. The findings are expected to provide technical and material support for the prevention and control of PEDV and PoRVA epidemics.

## 2. Materials and Methods

### 2.1. Collection and Pre-Treatment of Clinical Samples

A total of 7107 intestinal and fecal samples were collected from diseased piglets during the outbreak of diarrhea on immunized farms in Henan, Hubei, Jiangsu, Shandong, Guangdong, and Hunan, Jiangxi, and Sichuan provinces of China from 2018 to 2021. The specific source information of the samples is presented in Table 1. The samples were frozen and thawed three times to release the virus after homogenization with phosphate-buffered saline (PBS, Gibco, Thermo Fisher Scientific, Waltham, MA, USA). After centrifugation at 15,000 rpm for 10 min, the supernatants were collected for the extraction of viral nucleic acid and the isolation of the virus.

### 2.2. Reverse Transcription PCR (RT-PCR)

The viral RNA was extracted by using MolPure^®^ Viral DNA/RNA Kit (Yeasen, Shanghai, China) following the manufacturer’s instructions. The quantity and quality of the extracted RNA were measured by using a Nanodrop spectrophotometer (Thermo, Waltham, MA, USA). Viral RNA was then transcribed into cDNA by utilizing reverse transcription using the HiScript II 1st Strand cDNA Synthesis Kit (Vazyme, Nanjing, China). The primers targeting the PEDV M gene, PDCoV M gene, PoRVA VP7 gene, and TGEV N gene were designed and shown in Table 2. The cDNA was screened by RT-PCR using the 2 × Taq Master Mix (Dye Plus) (Vazyme, Nanjing, China) on SimpliAmpTM Thermal Cycler (Thermo Fisher Scientific, Waltham, MA, USA).

### 2.3. Sanger Sequencing

For amplification of the PEDV S gene and ORF3 gene, the primers against the S1, S2, and ORF3 genes were designed by using Primer 5.0. The sequences of primers are shown in Table 3. Then, the cDNA of 96 clinical samples and five isolated PEDV strains was used to amplify the PEDV S1, S2, and ORF3 genome by using 2 × Phanta Max Master Mix (Dye Plus) (Vazyme, Nanjing, China). The PCR products were observed and acquired under ultraviolet after electrophoresis in 0.01–0.03% YeaRed (Yeasen, Shanghai, China) stained agarose gel. Subsequently, the PCR products were extracted by E.Z.N.A.^®^ Gel Extraction Kit (OmegaBio-tek, Inc., Norcross, GA, USA) and sent to Tsingke Biotechnology Co., Ltd. (Beijing, China) for sanger sequencing.

### 2.4. Phylogenetic and Evolution Analysis

A total of 213 PEDV S gene sequences, 208 PEDV ORF3 gene sequences, 59 VP7 gene sequences of human RVA strains, and 61 VP7 gene sequences of PoRVA strains uploaded after 2010 in China were downloaded from GenBank. In addition, the PEDV S1 gene of 96 clinical samples were sequenced. The genomic sequences of each gene were aligned by using MAFFT v7.4.02 [19], respectively. The phylogenetic tree was generated by the neighbor-joining method in MEGAX software with a p-distance model, using 1000 bootstrap replicates [20]. Potential recombination events in the complete genomes of PEDV DY2020 strains isolated in this study and other PEDV strains were assessed by the Recombination Detection Program v4.39 (RDP4), which included nine detection algorithms (RDP, GENECONV, Bootscan, Maxchi, Chimaera, SiSscan, PhylPro, LARD, and 3Seq) [21].

### 2.5. Cell Culture and Virus Isolation

Vero-E6 cell lines (Purchased from ATCC, Manassas, VA, USA) were cultured in Dulbecco’s modified Eagle medium (DMEM, Gibco, Thermo Fisher Scientific, Waltham, MA, USA) supplemented with 10% heat-inactivated fetal bovine serum (FBS, Natocor, Cordoba, ARG), and antibiotics (100 units/mL of penicillin, 100 μg/mL of streptomycin, and 0.25 μg/mL of Fungizone^®^, Thermo Fisher Scientific, Waltham, MA, USA), at 37 °C in a humidified incubator containing 5% CO_2_.

For PEDV isolation, 90%-confluent Vero cell monolayers were washed with PBS twice. Next, 200 μL of inoculum per well for a 6-well plate was added. After incubation at 37 °C for 2 h, 2 mL of PEDV growth medium (DMEM supplemented with antibiotics, and 10 μg/mL of trypsin (Gibco, Thermo Fisher Scientific, Waltham, MA, USA) was added with the removal of the inoculum. After 48–72 h, the plate was frozen at −80 °C and thawed twice. The cells and supernatants were mixed by pipetting and stored at −80 °C. These samples were used as seed stocks for the next passage. For serial passaging, the culture scale was gradually increased, until finally T75 flasks were used for propagation and serial passage of PEDV strains. Virus RNA was extracted every 5 passages for RT-PCR detection of the virus nucleic acids.

### 2.6. Viral Growth Kinetics

The growth kinetics of isolated PEDV strains was determined by using a one-step growth curve and the 50% tissue culture infective dose (TCID_50_) assay. Monolayers of Vero cells were inoculated with isolated PEDV strains at a volume ratio of 1:10. After 2 h, the monolayers were washed twice with PBS, and 2  mL of DMEM containing 2% FBS was added. The culture supernatant was collected at 0, 2, 6, 12, 18, 24, 30, and 36 h post-inoculation and was then used to calculate the TCID_50_ of the virus after being frozen at −80 °C and thawing twice.

For TCID_50_ determination of PEDV strains, Vero cell monolayers in 96-well plates were inoculated with the culture supernatants of each strain serially diluted in DMEM at 37 °C for 1.5 h. Then, the infected cells were cultured in DMEM supplemented with 10% heat-inactivated FBS at 37 °C with 5% CO_2_ for 48 h. The cytopathic effect (CPE) on Vero cells was determined to calculate the TCID_50_ based on the Reed–Muench formula [21].

### 2.7. Swine Enteric Virus Investigation

The positive rate of four swine enteric viruses from 2018 to 2021 was used to calculate the co-infection rate of each virus in the positive samples. According to the climate in south-central China, the 12 months of the year were divided into four seasons, namely spring (March to March), summer (June to August), autumn (September to November), and winter (December to February), and statistical analysis was performed on the positive rate of PEDV in different seasons to determine the seasonality of PEDV infection. By referring to the conserved sequences of the PEDV reference strains, Primer 5.0 was used to design primers for the main virulence genes of PEDV (S gene and ORF3 gene) and PoRVA (VP4, VP6, and VP7 genes).

### 2.8. Statistical Analysis

All the data were analyzed using GraphPad Prism 8.0.1 software (GraphPad Software Inc., La Jolla, CA, USA), and presented as means ± standard error of the mean (SEM). The student’s t-test was used for difference analysis between groups, and *p* < 0.05 was considered a significant difference. All experiments were repeated more than thrice [22].

## 3. Results

### 3.1. Positive Rate of Swine Enteric Viruses from 2018 to 2021

**Figure 1:**
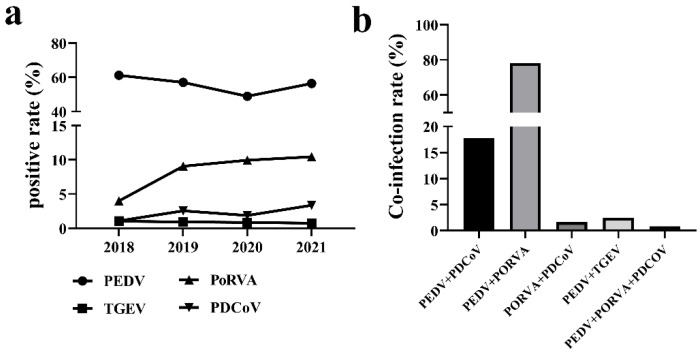
**Positive rate of swine enteric viruses from 2018 to 2021.** (**a**) Variation trend of the positive rate of diarrhea virus from 2018 to 2021. The *X*-axis represents the year, and the *Y*-axis represents the positive rate of PEDV, TGEV, PoRVA, and PDCoV. (**b**) Co-infection rate of swine enteric viruses in samples collected from 2018 to 2021. The *X*-axis represents the pattern of co-infection, and the *Y*-axis represents the co-infection rate.

RT-PCR assay was performed to detect PEDV, PDCoV, PoRVA, and TGEV in the intestinal and fecal samples collected from 2018 to 2021. The detection rate of each virus was statistically analyzed and represented by a line graph. The results are shown in Table 4 and Figure 1a. PEDV was found to be the dominant cause of diarrhea in piglets, whose average positive rate was 56.09% during the investigation period. The positive rate of PEDV showed a sustained decreasing trend from 2018 to 2020. Surprisingly, it increased and reached 56.44% (740/1311) in 2021. PoRVA was the second major cause of diarrhea in piglets, whose positive rate increased gradually, reaching 10.45% (137/1311) in 2021. TGEV and PDCoV were the minor causes of diarrhea and remained at low levels, but the positive rate of PDCoV showed a slight increase in 2021.

The information of all PCR positive samples was used to determine the co-infection of PEDV, PoRVA, TGEV, and PDCoV. As shown in Figure 1b, a total of 125 samples were infected by multiple enteric viruses. About 77.6% (97/125) of PEDV positive samples were found to be co-infected by PoRVA, followed by 17.6% (22/125) by PDCoV and 2.4% (3/125) by TGEV. The positive rate of PoRVA and PDCoV co-infection was 1.6% (2/125). The co-infection of PEDV, PoRVA, and PDCoV was extremely rare, accounting for only 0.8% (1/125).

### 3.2. Seasonal Characteristics and Distribution of PEDV

**Figure 2:**
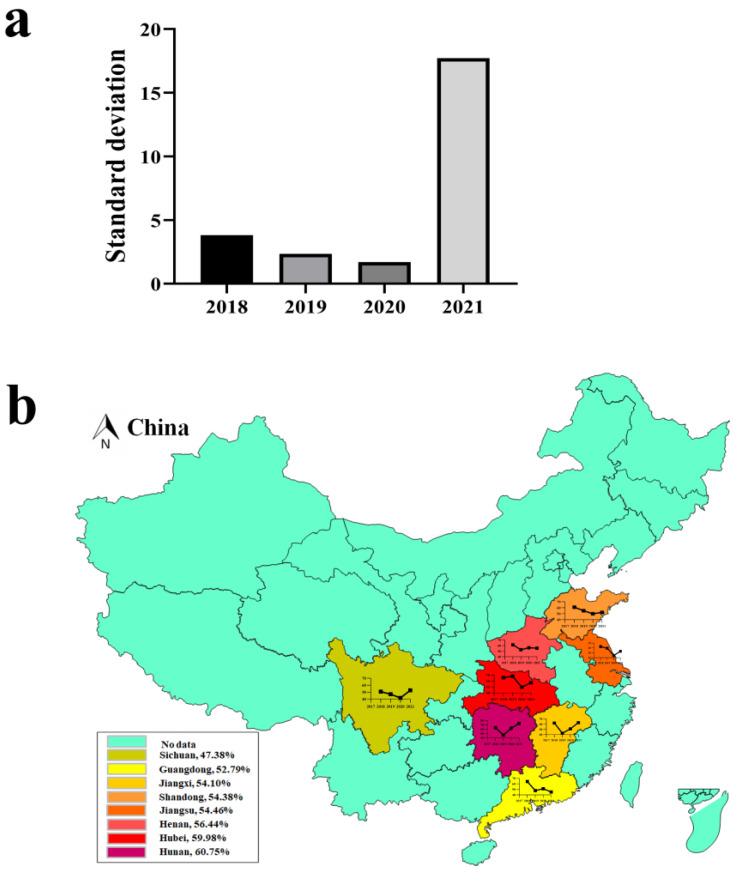
**Seasonal characteristics and distribution of PEDV.** (**a**) The standard deviation of PEDV in samples collected in different seasons from 2018 to 2021. The *X*-axis represents the year, and the *Y*-axis represents the standard deviation of the PEDV positive rate in the four seasons from 2018 to 2021. (**b**) Distribution of PEDV detection rate in eight major pork-producing provinces of China from 2018 to 2021. The yellow area indicates a low positive rate from 2018 to 2021; the red area indicates the highly endemic areas; a darker color indicates a higher positive rate. Percentages represent the overall positive rate from 2018 to 2021. The histogram indicates the positive rate in each province, and the point indicates the positive rate of PEDV in that year (Note: the map does not represent the true borders of administrative regions of China).

According to the climatic conditions of south-central China, the 12 months were divided into four seasons, namely spring (March–May), summer (June–August), autumn (September–November), and winter (December–February). The information of all PCR positive samples was used to calculate the positive rate of PEDV for each reason. As shown in Table 5, the positive rate of PEDV reached the highest in spring and winter, which was 71.79% (313/436) and 58.76% (191/325), respectively, while it was relatively low in the other two seasons, with the lowest level of 36.88% (97/263). The standard deviation of the PEDV positive rate in the four seasons from 2018 to 2021 was calculated. As shown in Figure 2a, the standard deviation from 2018 to 2021 was 3.81, 2.35, 1.70, and 17.72, respectively. The seasonality for the prevalence of PEDV tended to decrease from 2018 to 2020, and high positive rates may be present in any season in south-central China, however, the standard deviation in winter and spring in 2021 increased significantly, which may be due to the PEDV pandemic.

We identified the incidence of PEDV infection in major pork-producing provinces in China from 2018 to 2021. As shown in Figure 2b, the incidence of PEDV infection varied in different provinces of China. Cases occurred in south-central China provinces, like Henan, Hubei, Jiangsu, Shandong, Guangdong, Hunan, Jiangxi, and Sichuan, with the overall incidence of 56.44% (548/971), 59.98% (523/872), 54.46% (409/751), 54.38% (466/857), 52.79% (444/841), 60.75% (568/935), 54.10% (429/793), and 47.38% (515/1087). In 2018, 2019, 2020, and 2021, the provinces with the highest PEDV positive rate were Hubei (65.70%), Hubei (68.20%), Hunan (61.89%), and Hunan (73.10%), respectively. These results revealed that the provinces of central China, such as Hunan and Hubei, are the most severely affected areas by PED, while Sichuan, the largest province of pig production in China, has the lowest detection rate of PEDV.

### 3.3. Phylogenetic and Recombination Analysis of PEDV

**Figure 3:**
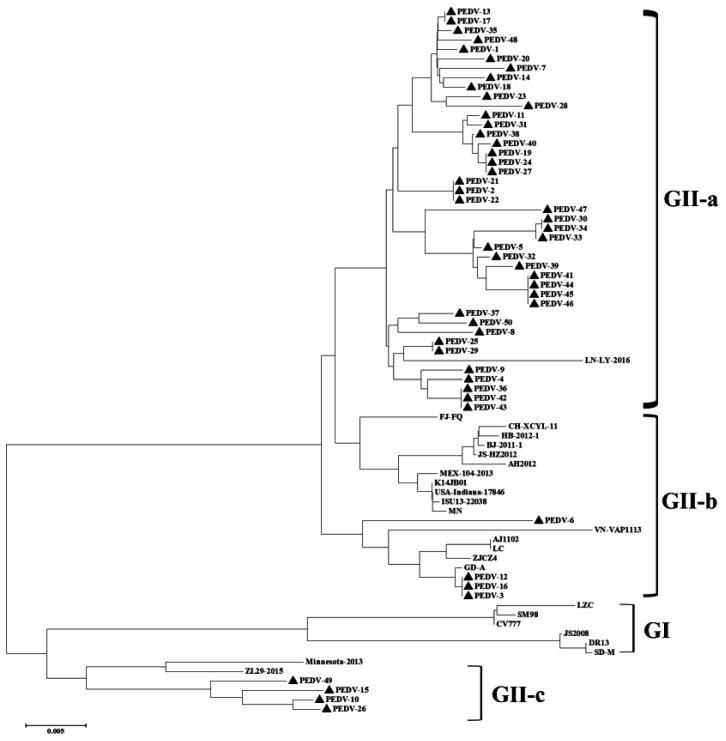
**Phylogenetic analysis based on the PEDV S1 genes sequenced in this study.** The phylogenetic tree was generated by the neighbor-joining method in MEGAX software with a p-distance model, using 1000 bootstrap replicates. The triangles represent the strains isolated in this study, and those without triangles represent the reference strains.

The S gene is the main virulence gene of PEDV, whose mutation leads to significant variations in PEDV virulence. A genetic evolution analysis was performed on the reference strains within each gene subgroup of PEDV, and the S1 gene sequences of 50 clinical samples were determined in this study. As shown in Figure 3, the S1 genes in this study all belonged to the new variant strain of PEDV, with 43 falling into the GII-a subgroup, four belonging to the GII-b subgroup, and three belonging to the GII-c subgroup. 

**Figure 4:**
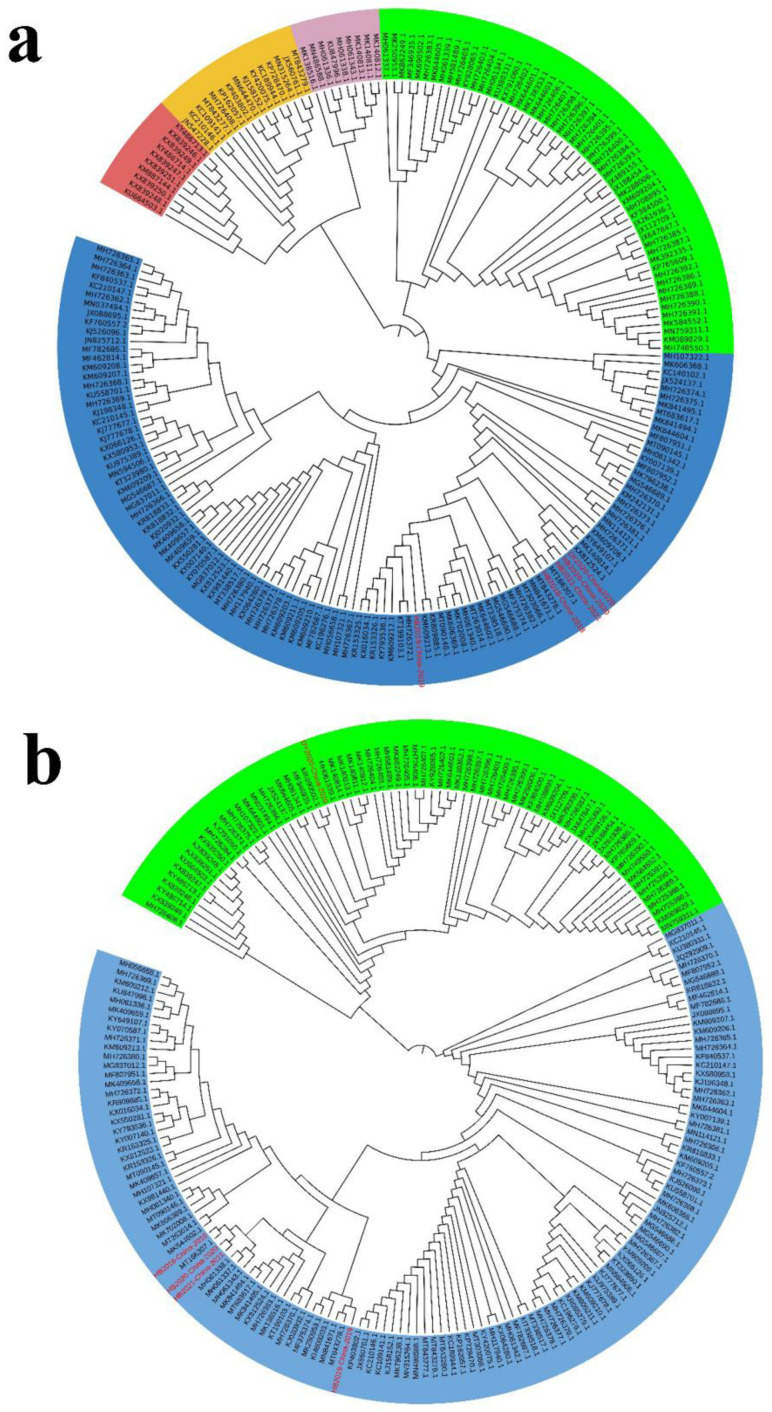
**The phylogenetic analysis based on the S genes and ORF3 genes of the PEDV strains isolated in this study.** (**a**) The phylogenetic analysis of the S gene. PEDV was divided into 5 categories: GII-a (Blue), GII-b (Green), GII-c (light pink), GI-a (light red), and GI-b (Yellow). The strains isolated in this study are highlighted in red color. (**b**). The phylogenetic analysis of the ORF3 gene. PEDV was divided into two categories: A group (Blue) and B group (Green). The strains isolated in this study are highlighted in red color. The phylogenetic trees were generated by the neighbor-joining method in MEGAX software with a p-distance model, using 1000 bootstrap replicates.

The S genes of the five viruses isolated in this study were compared with the reference sequences. As shown in Figure 4a, the phylogenetic tree constructed based on the S gene genome revealed that the PEDV strains in China could be mainly divided into two major groups (GI group and GII group). The GI group mainly included classic PEDV strains and recombinant strains, while most of the PEDV epidemic strains in China emerging in recent years belong to the GII group (variant strains). The strains of the GII group can be divided into two subgroups, GII-a and GII-b. In recent years, the strains of the GII-a subgroup have increased rapidly and tend to become a dominant subgroup. The five isolates isolated in this study were in different branches from the classic strain CV777, and all belonged to the GII-a subgroup, among which DY2020, HB2020, and HB2021 were located in the same branch.

ORF3 gene is an important virulence gene of PEDV. We compared the ORF3 gene sequenced in this study with the ORF3 gene sequences of PEDV strains isolated from multiple regions in China and constructed a phylogenetic tree. As shown in Figure 4b, the PEDV strains were clustered into two groups based on the ORF3 gene, namely the A group and B group. The A group mainly included part of members in the GI group and GII-a subgroup, while the B group mainly comprised part of members in the GI group and GII-b subgroup. The ORF3 gene of the HB2018, HB2019, HB2020, and HB2021 strains was in the same branch as the GII-a strain, and that of the DY2020 strain was in the same branch as the GII-b subgroup strain, indicating that there is gene recombination between strains in the GII-a subgroup and GII-b subgroup.

**Figure 5:**
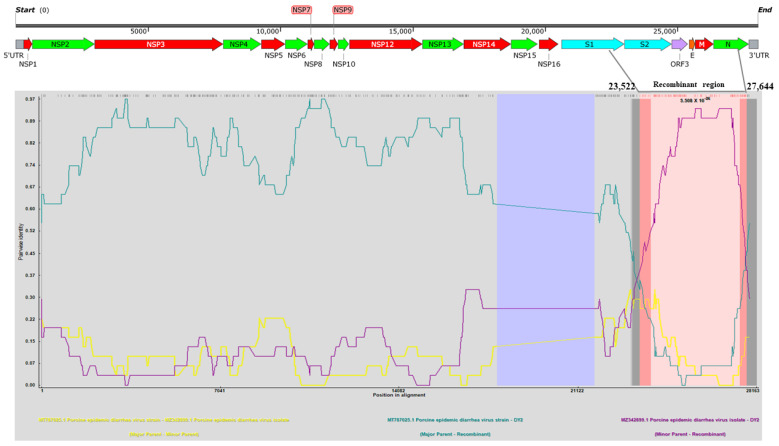
**The recombination analysis of the complete genome of PEDV DY2020 strains isolated in this study.** The results were described using the RDP method, which was supported by ≥6 programs to further characterize the potential recombination events. The pink box indicates the regions for the occurrence of recombination events. The *Y*-axis represents the pairwise identity between the recombinant and its putative parents. The *X*-axis represents the position in alignment with a 30 nt sliding window. The comparison of the recombinant-major parent, recombinant-minor parent, and major-minor parent was indicated by cyan, purple, and yellow lines, respectively.

A complete genome recombination analysis was performed between the DY2020 strains and 125 reference strains by using RDP4 software. As shown in Figure 5, the PEDV DY2020 strain might arise from the recombination of MT787025.1 (major parent, GII-a) and MZ342899.1 (minor parent, GII-a), which was supported by six detection algorithms (RDP, *p*-values ≤ 1.121 × 10^−27^; GENECONV, *p*-values ≤ 2.441 × 10^−25^; Maxchi *p*-values ≤ 1.125 × 10^−13^; Chimaera, *p*-values ≤ 5.111 × 10^−15^; SiSscan, *p*-values ≤ 2.409 × 10^−16^; 3Seq, *p*-values ≤ 1.374 × 10^−35^). Further analysis indicated that the position 23,522–27,644 of the complete genome of the DY2020 strain was the predicted genetic recombination fragment, and the identified putative breakpoints were located in the S gene, ORF3 gene, and the genes encoding envelope protein, membrane protein, and part of nucleocapsid proteins of the PEDV DY2020 strain.

### 3.4. Isolation and Titer Analysis of PEDV

**Figure 6:**
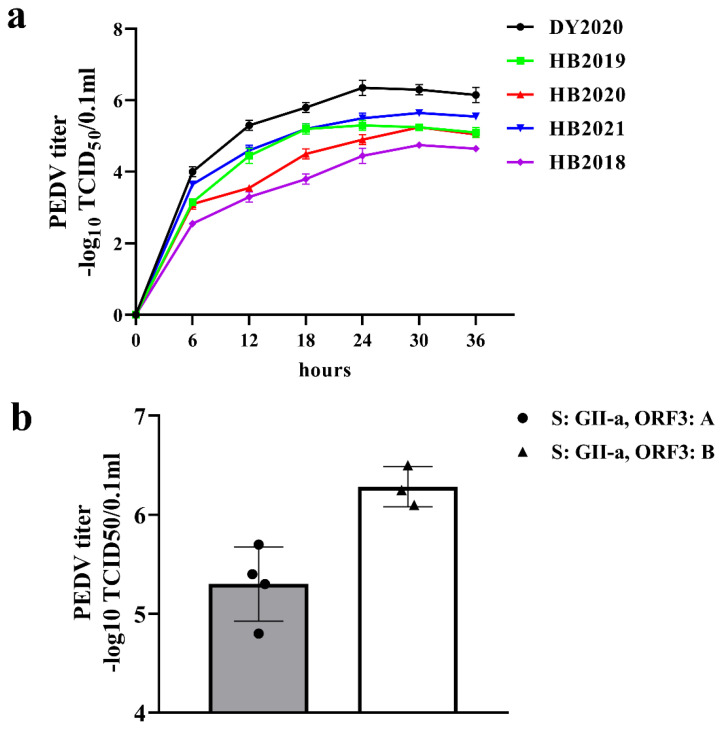
**The growth kinetics of isolated PEDV strains and titer analysis**. (**a**) Growth curves of isolated PEDV strains in Vero cells. (**b**) The relationship between PEDV strain titer and ORF3 gene clustering. The dots represent HB2018, HB2019, HB2020, and HB2021 strains, and the triangles represent DY2020, HM2017, and CH/HLJ/18 strains [23,24].

The growth kinetics of the five isolated PEDV strains was evaluated by measuring TCID_50_ at different infection time points. As shown in Figure 6a, the PEDV titers showed significant time-dependent increases and the DY2020 strain had the fastest growth in the first 12 h after infection. The titers of PEDV DY2020 strains were significantly higher than those of the other four PEDV strains at each infection stage, reaching a peak value of 10^6.2^ TCID_50_/0.1 mL at 24 h post-infection. The phylogenetic analysis based on the S gene of PEDV strains isolated in this study revealed that the DY2020 strain and the other four strains belonged to the GII-a subgroup. However, based on ORF1gene, the other 4 strains belonged to the A group, and the DY2020 strain belonged to the B group, which was similar to the case of HM2017 and CH/HLJ/18 strains [23,24]. The growth characteristics of HM2017 and CH/HLJ/18 strains were similar to those of DY2020 isolates (Figure 6b).

### 3.5. Phylogenetic Analysis of PoRVA

**Figure 7:**
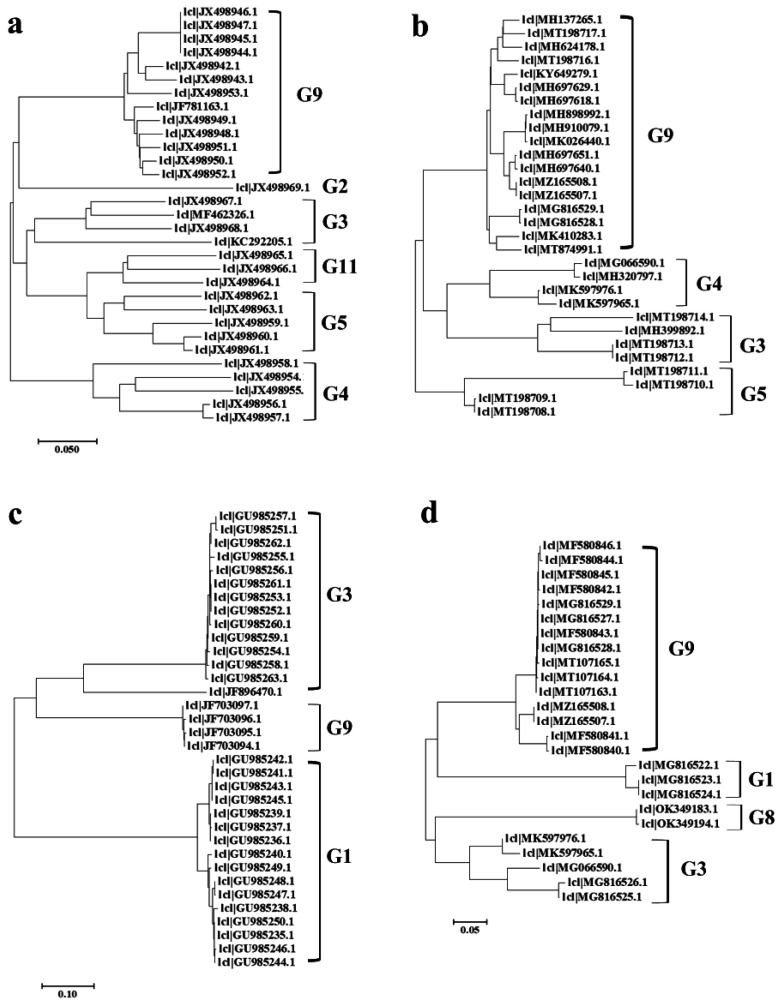
**Phylogenetic analysis based on the VP7 genes of the PoRVA strains and human rotavirus strains.** (**a**,**b**) Phylogenetic analysis based on the VP7 genes of the PoRVA strains before (**a**) and after (**b**) 2018. (**c**,**d**) Phylogenetic analysis based on VP7 genes of human rotaviruses strains before (**c**) and after (**d**) 2018.

The G genotype of PoRVA is determined by its VP7 gene [11]. VP7 nucleotide with homology higher than 80% can be identified as the same genotype (G type). The phylogenetic analysis based on VP7 genes of porcine PoRVA strains demonstrated that G9 is always the most common G genotype of PoRVA in China (Figure 7a,b). The phylogenetic analysis based on VP7 gene of human rotaviruses strains showed that before 2017, human rotaviruses in China were mainly G1 and G3 types, while the G9 type only accounted for a small proportion. However, after 2018, the G9 type strain became the dominant genotype of human rotavirus (Figure 7c,d).

## 4. Discussion

Piglet viral diarrhea is an important cause of piglet death, leading to serious production reduction and huge economic loss in the pork industry. PEDV, PoRVA, PDCoV, and TGEV are the most common pathogens causing piglet diarrhea [25,26]. In this study, a total of 7107 diarrhea-related samples collected from pig farms in south-central China from 2018 to 2021 were examined to identify the major diarrhea pathogens. As a result, PEDV was identified as the most important cause of diarrhea, and its detection rate decreased from 2018 to 2020, which may be due to the wide use of the PEDV vaccine (GII-b) in China [18,27,28,29]. In addition, in 2018, the outbreak of African swine fever (ASF) in pigs was first reported in China, and was transmitted rapidly through the legal trade of live pigs, which led to the development of ASFV monitoring and control plans and the upgrading of the biosecurity system of pig farms [30,31,32]. These may be important reasons for the decline in the PED positive rate after 2018, and also provide some useful data for pig farms and the government to develop a bio-safety system for more efficient prevention and control of infectious diseases. In 2019, the severe acute respiratory syndrome coronavirus 2 (SARS-CoV-2) was first reported in Wuhan city of China, causing the coronavirus disease 2019 (COVID-19). As a result, strict nationwide prevention and control of COVID were implemented in China, which significantly restricted the flow of pedestrian, logistics, and animal products [33,34]. Due to the impact of COVID-19, the number of samples collected each year was inconsistent between 2018 and 2021, with the smallest number in 2021. However, our results still showed that the detection rate of PEDV increased sharply in 2021, indicating the potential risk of another outbreak and therefore the need to develop new PEDV vaccine strains. In addition, the detection rate of TGEV has been at a low level without a trend of large-scale epidemics, while the detection rate of PoRVA and PDCoV have had a rising tendency in recent years. The detection results of co-infection revealed that PEDV and PoRVA co-infection is currently the most important form of co-infection rather than the previous PEDV and TGEV co-infection [35,36], indicating that the monitoring and prevention of PoRVA should be strengthened. The seasonality for the prevalence of PEDV has been gradually decreasing [37,38], and many provinces showed high incidence throughout the year, suggesting that the prevention and control of the epidemic should be strengthened in the summer and autumn when PEDV was low in the past. Furthermore, we found that Hunan and Hubei were the provinces with the most severe PEDV epidemics in south-central China, which is consistent with the study of He et al. [39]. In addition, our results revealed that the GII-a subgroup has been increasing rapidly and has become dominant in recent years. The PEDV epidemic has been circulating many times and there is a trend of re-emergence [40,41,42]. Therefore, more attention should be paid to the PEDV GII-a subgroup [43]. The epidemiological investigation and research on the latest PEDV variants will provide theoretical guidance for the prevention and control of PED and provide vaccine reserves for preventing the outbreak of PED epidemics.

In this study, five PEDV variants were isolated from diarrhea samples using a Vero-E6 cell line, and primers were designed to sequence their main virulence genes (S gene and ORF3 gene) [18]. According to the genetic evolution analysis of the S gene, these five PEDV strains belong to the GII-a subgroup, which is closely related to the PEDV variant strains isolated in China in recent years, and possesses relative low homology to the commercial vaccine strain CV777 [44,45,46,47,48,49]. According to the genetic evolution analysis of the ORF3 gene, other four PEDV strains, except for DY2020 isolated in this study, are in the same branch as the strains of the GII-a subgroup, and these four strains form a small branch independently with the PEDV variant strains isolated in China in recent years, which may be caused by mutations at multiple loci. In addition, the ORF3 gene of one PEDV variant strain was in the same branch as that of the GII-b subgroup, indicating that the strains of the GII-a subgroup and the GII-b subgroup of PEDV might have undergone recombination in the natural environment. In addition, the recombinant DY2020 strain exhibited earlier and more obvious CPE on Vero cells, suggesting that mutation in the ORF3 protein may have altered its infectivity [23,24], which needs further verification. A comparison of the nucleotide and amino acid homology between the five strains and the commercial vaccine strains revealed that they have rather low homology. In recent years, the re-emergence of PEDV may be caused by these genetic variants [18]. Further research on the variant strains will help to understand the mechanisms for the re-emergence of PEDV and help the prevention of PED.

The gene sequences of PEDV epidemic strains at present are significantly different from those of previous classic strains and vaccine strains, and the nucleotide sequences of the S gene and ORF3 gene are also significantly different from those of PEDV epidemic strains and the classic strains CV777 reported previously [18]. Since 2010, large-scale PED outbreaks have frequently occurred in China due to the variation of PEDV. In addition, PEDV tends to have continuous mutations, posing new challenges to the prevention and control of PED. It is therefore necessary to strengthen the epidemiological study of PEDV mutant strains and pay close attention to the genetic variation of the pathogens. There are great differences between PEDV and existing commercial vaccine strains, and more PEDV mutant strains are emerging [43,50]. Therefore, China is in urgent need for vaccine strains corresponding to the epidemic strains, and research on the characteristics of epidemic strains will provide technical reserves for the control of PED epidemics.

The prevalent PoRVA G genotypes mainly include G9, G11, G2, G3, G4, and G5, among which G5 and G9 are the main genotypes in China [51]. The wide application of commercial vaccines, such as the TGEV-PEDV-PoRVA (G5) triple live vaccine, has effectively controlled the epidemic of the PoRVA G5 genotype in China. However, at the same time, we found that more G9-type PoRVAs were isolated from piglet diarrhea samples in China and other regions of Asia, which also demonstrates that G9-type PoRVA has been increasing in China and other Asian countries and has become the most predominant G type [52,53]. The G5 strains of PoRVA may be gradually reduced under the selection pressure of PoRVA (G5) vaccines, while the G9 strains slowly emerged through the results of epidemiological investigations. The typical sequences of the VP7 gene of human rotavirus strains (59) and pig PoRVA strains (61) from 2010 to 2021 were downloaded from GenBank. The evolutionary analysis revealed that rotavirus G1 and G3 strains were dominant before 2017, while after 2018 there was a surge of human G9 strain. The G9 strain, meanwhile, has been present and dominant in swine and dogs [51,54]. Previous studies have revealed that the G3 and G9 strains of RAV cause diarrhea in human infants and young children, and the recombination of RVA in humans and animals causes the generation of new PoRVA genotypes [55,56,57,58,59]. Therefore, we hypothesize that the wide application of related vaccines may largely reduce the prevalence of human G1 and G3 and pig G5 genotypes, while the rotavirus G9 genotype will eventually become the dominant genotype, which will undoubtedly increase the pressure on the prevention and control of porcine rotavirus. In addition, the G9 genotype also has the risk of infecting infants and young children, and thus calls for more attention [51,57,60,61,62]. By analyzing the prevalence of PoRVA and human rotavirus in China and studying the genetic evolution relationship of its genotypes, this study facilitates a better understanding of the trend of the G9 subtype and provides some important implications for the control of the rotavirus epidemic.

## Figures and Tables

**Table 1:** The information of the samples collected from each province from 2018 to 2021.

Year	Number of Samples							
	Henan	Hubei	Jiangsu	Shandong	Guangdong	Hunan	Jiangxi	Sichuan
2018	241	242	201	220	231	257	211	298
2019	225	217	177	211	203	221	197	271
2020	317	266	232	259	254	286	237	322
2021	188	147	141	167	153	171	148	196

**Table 2:** Primers for swine enteric virus gene detection.

Primer Name	Sequence	Product Length	Target Gene
PDCoV-P1	CCAGCAACCACTCGTGTTA	620 bp	M gene
PDCoV-P2	GTCCTTAGTTGGTTTGGTGGGT		
PEDV-P2	GTCTAACGGTTCTATTCCCG	460 bp	M gene
PEDV-P3	ATAGCCCTCTACAAGCAATG		
TGEV-P5	TTACAAACTCGCTATCGCATGG	528 bp	N gene
TGEV-P6	TCTTGTCACATCACCTTTACCTGC		
PORVA-P7	CCCCGGTATTGAATATACCACAGT	333 bp	VP7 gene
PORVA-P8	TTTCTGTTGGCCACCCTTTAGT		

**Table 3:** Primers for PEDV S gene and ORF3 gene sequencing.

Primer Name	Sequence	Product Length	Purpose
S1-F	ATGAAGTCTTTAACGTACTTCTGG	2208 bp	PEDV gene sequencing
S1-R	TAGAAGAAACCAGGCAACTCC		
S2-F	ATGCATTCTAATGATGGCTCTAAT	1953 bp	
S2-R	CTGCACGTGGACCTTTTCAAAAAC		
ORF3-F	ATGTTTCTTGGACTTTTTCAGTACA	675 bp	
ORF3-R	ACTAATTGTAGCATACTCGTCTAG		

**Table 4:** Positive rates of swine enteric virus from 2018 to 2021.

Year	Positive Rate (%) and (Number of Positive Samples/Number of Samples)			
	PEDV	TGEV	PoRVA	PDCoV
2018	61.02 (1160/1901)	1.05 (20/1901)	4.00 (76/1901)	1.10 (21/1901)
2019	57.03 (982/1722)	0.93 (16/1722)	9.06 (156/1722)	2.56 (44/1722)
2020	50.81 (1104/2173)	0.87 (19/2173)	9.94 (216/2173)	1.89 (41/2173)
2021	56.44 (740/1311)	0.76 (10/1311)	10.45 (137/1311)	3.36 (44/1311)

**Table 5:** Positive rates of swine enteric virus from 2018 to 2021.

Year	Positive Rate (%) and (Number of Positive Samples/Number of Samples)			
	Spring	Summer	Autumn	Winter
2018	63.14 (322/510)	55.56 (210/378)	59.34 (244/411)	63.79 (384/602)
2019	57.54 (271/471)	54.76 (190/347)	54.86 (209/381)	59.66 (312/523)
2020	52.87 (276/522)	49.28 (239/485)	49.60 (249/502)	51.20 (340/664)
2021	71.79 (313/436)	36.88 (97/263)	48.43 (139/287)	58.76 (191/325)

## Data Availability

The datasets used and/or analyzed during the current study are available from the corresponding author on reasonable request.
